# Predicting Cognitive Impairment in Elderly Patients with HFpEF: Development of a Simple Clinical Risk Score

**DOI:** 10.3390/jcm14113768

**Published:** 2025-05-28

**Authors:** Sergiu-Florin Arnautu, Brenda-Cristiana Bernad, Istvan Gyalai Korpos, Mirela-Cleopatra Tomescu, Minodora Andor, Catalin-Dragos Jianu, Diana-Aurora Arnautu

**Affiliations:** 1 Centre for Cognitive Research in Neuropsychiatric Pathology (NeuroPsy-Cog), Department of Neurosciences, “Victor Babes” University of Medicine and Pharmacy, E. Murgu Sq., no.2, 300041 Timisoara, Romania; arnautu.sergiu@umft.ro (S.-F.A.); jianu.dragos@umft.ro (C.-D.J.); 2 Multidisciplinary Heart Research Center, Department of Internal Medicine I, Faculty of Medicine, “Victor Babes” University of Medicine and Pharmacy, 2nd Eftimie Murgu Square, 340001 Timisoara, Romaniaaurora.bordejevic@umft.ro (D.-A.A.); 3 Doctoral School, “Victor Babes” University of Medicine and Pharmacy, E. Murgu Sq., no.2, 300041 Timisoara, Romania; 4 Center for Neuropsychology and Behavioral Medicine, “Victor Babes” University of Medicine and Pharmacy, 300041 Timișoara, Romania; 5 Cardiology Clinics, Timisoara Clinical Municipal Emergency Hospital, Bd. Revolutiei din 1989, no.12, 300041 Timisoara, Romania; 6 Neurology Clinics II, County Clinical Emergency Hospital, 156 L. Rebreanu Ave., 300736 Timisoara, Romania

**Keywords:** heart failure with preserved ejection fraction, cognitive impairment, elderly patients, cognitive risk score

## Abstract

**Background/Objectives:** Cognitive impairment is a frequent and underrecognized comorbidity in elderly patients with heart failure with preserved ejection fraction (HFpEF), contributing to poor outcomes and complicating disease management. This study aimed to identify risk factors associated with cognitive impairment in elderly HFpEF patients from Western Romania and to develop a point-based risk score for clinical use. **Methods:** We conducted a cross-sectional analysis of HFpEF patients aged ≥65 years. Cognitive status was assessed using the Mini-Mental State Examination-2 (MMSE-2), with significant impairment defined as a score <24. Multivariable logistic regression analysis was performed to identify independent predictors of cognitive dysfunction. **Results:** A total of 326 HFpEF patients were included. Diabetes mellitus, prior stroke or transient ischemic attack (TIA), carotid artery disease, elevated N-terminal pro–B-type natriuretic peptide (NT-proBNP), and reduced estimated glomerular filtration rate (eGFR) were independently associated with cognitive impairment. Higher Kansas City Cardiomyopathy Questionnaire (12-KCCQ) scores and anticoagulant therapy for atrial fibrillation were associated with a lower risk. Based on these variables, a simple point-based cognitive risk score was developed, demonstrating strong discriminatory ability (area under the curve = 0.84). A threshold of ≥2 points identified cognitive impairment with 75% sensitivity and 83% specificity. **Conclusions:** Our findings underscore the importance of integrated cardiovascular and cognitive assessment in elderly HFpEF patients. The developed risk score offers a pragmatic tool for the early identification of cognitive dysfunction, potentially informing timely interventions and preventive strategies.

## 1. Introduction

Heart failure (HF) is considered a global pandemic, currently affecting an estimated 64 million people worldwide. This number is projected to rise, primarily due to the aging population [1]. Although survival rates among HF patients have improved, mortality remains high [2,3,4].

One potential contributing factor to poor outcomes in HF patients is cognitive impairment [5,6]. A significant proportion of HF patients exhibit cognitive impairment, which is defined as a measurable deficit in one or more cognitive domains—positioned between normal cognitive functioning and dementia [7,8]. Prevalence estimates vary widely, from 25% to 75%, depending on the clinical context and assessment tools used [9,10,11].

Heart failure is closely linked to cognitive impairment through multiple interconnected mechanisms. Chronic cerebral hypoperfusion due to reduced cardiac output can lead to structural brain changes, including white matter lesions and hippocampal atrophy [12,13,14]. Systemic endothelial dysfunction and impaired blood–brain barrier (BBB) integrity promote neuroinflammation and neuronal injury. BBB disruption is associated with neurodegeneration and amyloid accumulation, processes seen in Alzheimer’s disease [15]. Neurohormonal activation, particularly of the sympathetic nervous system and RAAS, exacerbates oxidative stress and impairs cognitive pathways. Elevated plasma norepinephrine and aldosterone have been correlated with impaired cognition [6]. Inflammatory cytokines such as TNF-α and IL-6, commonly elevated in HF, further disrupt neural function [16]. Additionally, atrial fibrillation contributes to silent cerebral infarctions [17], while comorbidities like diabetes and renal dysfunction, along with polypharmacy, compound the risk [18].

A specific subtype of HF—heart failure with preserved ejection fraction (HFpEF)—accounts for up to 55% of all HF cases. It is characterized by typical HF symptoms, an ejection fraction above 50%, and evidence of left ventricular diastolic dysfunction on echocardiography or cardiac catheterization [19]. With global population aging, the burden of HFpEF is expected to grow, especially in low- and middle-income countries.

Patients with HFpEF are generally older, more likely to be female, and tend to have multiple chronic comorbidities such as hypertension, diabetes mellitus, atrial fibrillation, anemia, and obesity [2]. HFpEF is also strongly associated with functional decline, frailty, and cognitive impairment, which tends to be more prevalent than in patients with reduced ejection fraction (HFrEF), [19]. These comorbidities—particularly hypertension, atrial fibrillation, and diabetes mellitus—are well-established risk factors for cognitive decline [20,21,22].

Cognitive impairment may signal more severe cardiovascular disease and is associated with poorer outcomes. It can also directly worsen prognosis by impairing the patient’s ability to adhere to medications, diet, lifestyle modifications, and follow-up schedules [12,23]. Studies show that cognitive dysfunction significantly increases the risk of hospitalization and mortality in HF patients. In HFpEF, cognitive impairment is linked with a 40% increased risk of all-cause hospitalization and a two-fold increase in cardiovascular mortality [24].

Cognitive dysfunction also hampers patients’ capacity to manage complex HF treatment regimens, including medication adherence, fluid restrictions, and symptom monitoring, thereby accelerating clinical deterioration. For example, De Jong et al. [25] reported that medication non-adherence was nearly twice as high among cognitively impaired HF patients. Cognitive decline further correlates with greater disability, reduced physical functioning, and higher rates of depression. The interplay between mental and physical health in HF highlights the need for integrated care strategies.

Understanding the profile of cognitive decline in this demographic could enable more effective screening and targeted interventions. However, regional studies from Eastern Europe, and Romania specifically, are scarce.

Our objective was to identify risk factors associated with MMSE-defined cognitive impairment in elderly HFpEF patients from Western Romania. We also aimed to derive a point-based risk score and an interactive tool for clinical use.

## 2. Materials and Methods

### 2.1. Study Design and Population

This observational, cross-sectional, population-based study included patients aged 65 years or older with a confirmed diagnosis of heart failure with preserved ejection fraction (HFpEF), who were consecutively enrolled from a cardiology outpatient registry at the Municipal Emergency Hospital Timișoara, Romania, between January 2023 and December 2024. The inclusion criteria for the study were (1) a left ventricular ejection fraction (LVEF) ≥ 50%, (2) New York Heart Association (NYHA) functional class II–IV symptoms, and (3) clinically stable status at the time of enrolment.

Patients were classified as having heart failure with preserved ejection fraction (HFpEF) based on contemporary guideline criteria [26], which included (1) a documented history of heart failure symptoms and signs (e.g., dyspnea, fatigue, exercise intolerance, or fluid retention), (2) an LVEF ≥ 50%, and (3) objective evidence of cardiac structural and/or functional abnormalities consistent with diastolic dysfunction or elevated left ventricular (LV) filling pressures, including elevated levels of natriuretic peptides (NT-pro-BNP (over 300 pg/mL in patients with sinus rhythm and over 600 pg/mL in those with AF)).

Participants were excluded from the study if they met any of the following conditions: left ventricular ejection fraction (LVEF) < 50%, indicating heart failure with reduced ejection fraction; recent acute cardiovascular events such as acute coronary syndrome or stroke; or uncontrolled severe hypertension defined as systolic blood pressure > 180 mmHg and/or diastolic blood pressure > 130 mmHg. Individuals with significant valvular heart disease, including severe mitral or aortic regurgitation, were also excluded. Additional exclusion criteria included a history of chronic alcohol or drug abuse; the presence of major psychiatric disorders such as schizophrenia, post-traumatic stress disorder, or paranoia; and individuals with severe depression or anxiety requiring active pharmacological treatment. Patients with a diagnosis of cognitive impairment who were already undergoing treatment were excluded to avoid confounding. Finally, individuals who were unable or unwilling to provide informed consent were not enrolled in the study.

### 2.2. Clinical and Laboratory Data Collection

At baseline, comprehensive data were collected for all participants, including demographic characteristics, relevant comorbidities (e.g., diabetes mellitus, atrial fibrillation), and cardiovascular history (e.g., stroke or transient ischemic attack, carotid artery disease). Information regarding chronic medication use was also documented.

All patients underwent a thorough clinical evaluation, including physical examination and vital signs assessment. Electrocardiography (ECG) was performed to assess for the presence of arrhythmias.

Cardiac structure and function were evaluated using transthoracic echocardiography (TTE) with a General Electric Vivid S5 ultrasound system. Standard two-, three-, and four-chamber views were acquired according to current guideline recommendations to assess left ventricular (LV) systolic and diastolic function and to calculate the left ventricular ejection fraction (LVEF) [27]. Common carotid artery (CCA) B-mode images were obtained for all patients using the same ultrasound system equipped with a multifrequency 7.5–10 MHz high-resolution vascular probe [28]. Carotid intima–media thickness (IMT) was measured at end-diastole in both CCAs, 1.0 cm proximal to the carotid bulb. The presence of carotid plaque was defined as an IMT ≥ 1.2 mm [29]. To ensure consistency, all echocardiographic and Doppler assessments were performed by the same experienced examiner using the same equipment.

Laboratory analyses included measurements of N-terminal pro–B-type natriuretic peptide (NT-proBNP), glycated hemoglobin (HbA1c), lipid profile, estimated glomerular filtration rate (eGFR), and serum uric acid. These parameters were used to evaluate metabolic and renal function, as well as to aid in the characterization of cardiovascular risk.

### 2.3. Functional and Cognitive Assessment

Functional capacity and health-related quality of life were evaluated using the short-version Kansas City Cardiomyopathy Questionnaire (12-KCCQ), a validated tool for quantifying the quality of life in patients with heart failure [30]. The predefined score categories are Poor (<25), Fair (25–<50, Good (50–<75), and Excellent (75 to 100).

Depressive symptoms were assessed using the Geriatric Depression Scale (GDS), a validated screening tool specifically designed for older adults. The short form of the GDS, consisting of 15 yes/no questions, was administered to all participants. A score of ≥5 points was considered indicative of possible depression, while higher scores corresponded to greater severity. The GDS was selected due to its simplicity, high sensitivity, and suitability for elderly populations with or without cognitive impairment [31].

Cognitive function was evaluated using the Mini-Mental State Examination, Second Edition (MMSE-2) [32]. The MMSE-2 was administered in person by a neurology specialist trained in the tool’s application. The test consists of 30 items assessing orientation, attention, memory, language, and visuospatial skills, with total scores ranging from 0 to 30. Higher scores indicate better cognitive function [33].

Although standardized cutoffs for the Mini-Mental State Examination (MMSE) have not been universally established, this study adopted thresholds consistent with prior literature to classify baseline cognitive function: scores less than 24 were indicative of cognitive impairment, scores ranging from 24 to 27 were considered reflective of borderline cognitive function, and scores between 28 and 30 were regarded as normal cognitive function [24,33,34,35,36]. Accordingly, the participants were categorized into three cognitive function groups based on MMSE scores: Group I, Normal cognition (MMSE score 28–30); Group II, Borderline cognitive impairment (MMSE score 24–27), and Group III, Definite cognitive impairment (MMSE score < 24).

### 2.4. Ethics Statement

The study protocol was reviewed and approved by the Institutional Review Board of the Municipal Emergency Hospital Timișoara, Romania (Approval No. I-29417/9 November 2022). All procedures were conducted in accordance with the ethical principles outlined in the Declaration of Helsinki. Written informed consent was obtained from all participants at the time of hospital admission, as mandated by the Romanian Health Authority.

### 2.5. Statistical Analysis

Baseline characteristics were summarized according to MMSE category and presented as frequency and percentage for categorical variables, mean ± standard deviation (SD) for normally distributed continuous variables, or median with interquartile range (IQR) for non-normally distributed variables. To assess trends across the three cognitive function groups (normal, borderline, and impaired), linear regression was used for parametric variables, and the Kruskal–Wallis trend test was applied to non-parametric variables.

Univariate logistic regression analyses were conducted to identify baseline characteristics associated with cognitive impairment, defined as an MMSE score < 24. Variables found to be significantly associated in univariate models were further assessed using multivariable logistic regression to determine independent predictors of cognitive impairment.

To evaluate the diagnostic performance of these independent predictors, a receiver operating characteristic (ROC) curve analysis was conducted to calculate sensitivity and specificity values. All statistical analyses were performed using MedCalc Statistical Software version 23.1.6 (MedCalc Software Ltd., Ostend, Belgium) [37]. A two-tailed *p*-value < 0.05 was considered indicative of statistical significance.

### 2.6. Risk Model Development and Performance Assessment

A predictive scorecard for cognitive impairment in HFpEF patients was developed using logistic regression modeling. Cognitive impairment was defined as a Mini-Mental State Examination-2 (MMSE-2) score < 24, serving as the binary outcome variable. Candidate predictors were selected based on clinical relevance and prior evidence, including demographic factors, comorbidities, biochemical markers, echocardiographic parameters, functional status, and medication use. Univariate logistic regression analyses were performed to assess the association of each variable with cognitive impairment. Variables with a *p*-value < 0.10 were retained for multivariable analysis. A stepwise multivariable logistic regression model was constructed to identify independent predictors, and multicollinearity was assessed to ensure model stability.

The β coefficients from the final multivariable model were used to assign relative weights to each predictor. Continuous variables were categorized into clinically meaningful thresholds, and a point-based score was calculated by rounding each coefficient to the nearest integer for ease of clinical use. Predictors associated with increased risk received positive scores, while those associated with decreased risk received negative scores. The total score for each patient was derived by summing the individual predictor points.

Model performance was evaluated using the area under the receiver operating characteristic curve (AUC) for discrimination, and calibration was assessed using the Hosmer–Lemeshow test. The resulting scorecard was intended to stratify patients into cognitive risk categories for potential clinical application.

## 3. Results

### 3.1. Baseline Characteristics

The study included 326 patients ≥ 65 years old, with a mean age of 73.4 ± 8.2, with 160 (49%) being men.

Table 1 presents the demographic and clinical characteristics of patients stratified by baseline MMSE score categories, with *p*-values indicating tests for linear trends across groups. Patients with lower MMSE scores were older and exhibited lower diastolic blood pressure, a poorer quality of life (lower 12-KCCQ scores), and a higher prevalence of diabetes, carotid artery disease, prior stroke or transient ischemic attack (TIA), depression, and chronic kidney disease. They also had significantly lower estimated glomerular filtration rates (eGFR) and elevated levels of total cholesterol, triglycerides, uric acid, hemoglobin A1c, and NT-proBNP. Additionally, patients with cognitive impairment were less frequently prescribed anticoagulants compared to those with preserved cognitive function.

**Figure 1:**
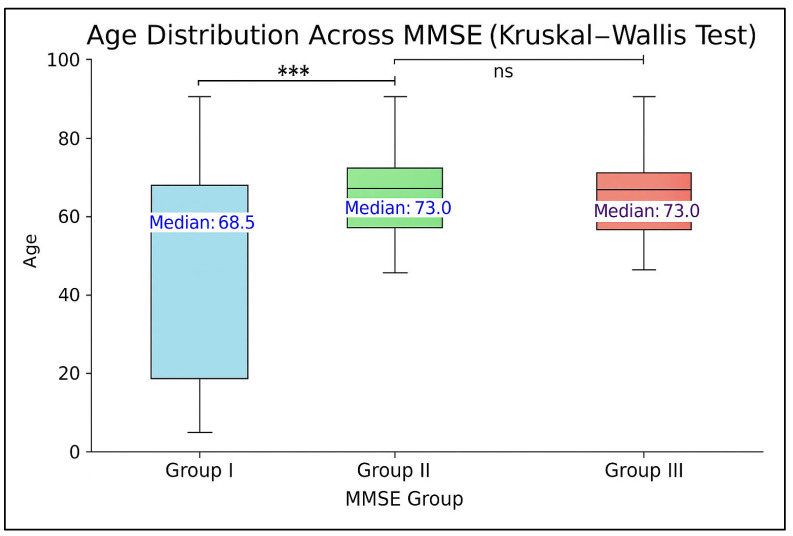
Boxplot showing the distribution of age according to Mini-Mental State Examination-2 (MMSE-2) score categories. The central line within each box indicates the median; the box limits represent the interquartile range (IQR); whiskers denote the range excluding outliers. Bonferroni-corrected *p*-values are displayed for each pairwise comparison between MMSE groups. *** = *p* < 0.001, ns = not significant.

The analysis of age distributions demonstrates a progressive increase in age across MMSE groups, supporting age as a relevant factor in cognitive decline among HFpEF patients, as shown in Figure 1. Pairwise comparisons revealed significantly higher age in Group II compared to Group I (*p* < 0.0001) and in Group III compared to Group I (*p* < 0.0001), while no significant difference was observed between Group II and Group III (*p* = 0.546).

Statistical comparisons were performed using the Kruskal–Wallis test, with Bonferroni-corrected *p*-values.

### 3.2. Predictors of Cognitive Impairment

A univariate regression analysis of the clinical, biochemical and echographic features of the HFpEF patients found that age, life quality evaluated by 12-KCCQ scale, comorbidities (systemic hypertension, diabetes mellitus, history of stroke/TIA, chronic kidney disease, carotid artery disease, depression), elevated total cholesterol, uric acid, triglycerides and NT-proBNP levels, and anticoagulant treatment for atrial fibrillation were significantly associated with an MMSE-2 score < 24 (Table 2).

Multivariable logistic regression included the variables identified in the univariate analysis and revealed several independent protective and risk predictors of cognitive impairment in HFpEF patients aged ≥65 years. We found the following protective predictors (OR < 1) for cognitive impairment: anticoagulant use for AF (strongly protective, OR = 0.03, *p* = 0.0017); higher 12-KCCQ score, indicating better quality of life (OR = 0.94, *p* = 0.0470), and higher estimated glomerular filtration rate, reflecting better kidney function (OR = 95, *p* = 0.0044). The risk predictors identified by OR > 1 were carotid artery disease (a strong risk factor, OR = 9.53, *p* = 0.0106); diabetes mellitus, associated with increased risk (OR = 5.47, *p* = 0.0095); history of stroke/TIA, associated with increased risk (OR = 5.99, *p* = 0.0349); and elevated NT-proBNP, associated with a modest but statistically significant increase in risk (OR = 1.0023, *p* = 0.0028), Table 3.

**Figure 2:**
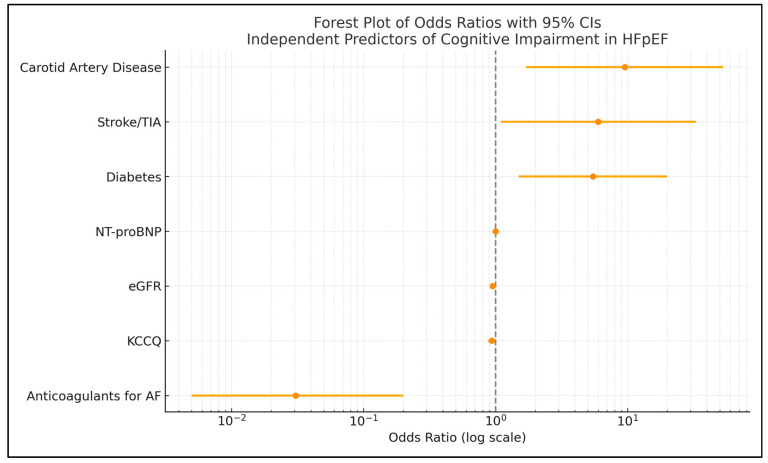
Forest plot of odds ratios with 95% confidence intervals of the independent predictors of an MMSE-2 score < 24, identified by multivariable logistic regression. Abbreviations: HFpEF, heart failure with preserved ejection fraction; AD, artery disease; TIA, transient ischemic attack; eGFR, estimated glomerular filtration rate; KCCQ, Kansas City Cardiomyopathy Questionnaire; MMSE, Mini-Mental State Examination; NT-proBNP, N-terminal pro–B-type natriuretic peptide; TIA, transient ischemic attack.

Figure 2 presents a Forest plot illustrating the odds ratios and 95% confidence intervals for the independent predictors of an MMSE-2 score below 24, as identified through multivariable logistic regression analysis.

**Figure 3:**
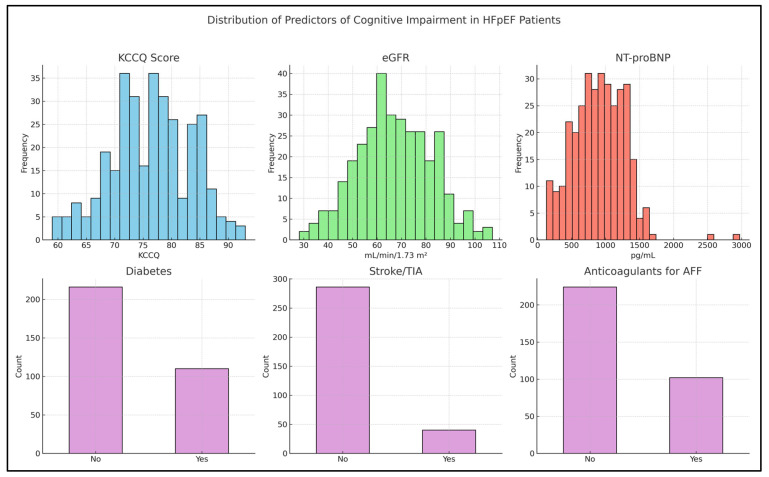
The distribution of the key predicting factors among the HFpEF patients. Histograms (top row) display the distribution of continuous variables: KCCQ score, eGFR, and NT-proBNP. KCCQ scores are generally high, with most patients scoring above 60, indicating preserved quality of life. eGFR values show a moderate spread, reflecting variation in renal function across the cohort. NT-proBNP levels exhibit a right-skewed distribution, consistent with clinical variability observed in heart failure. Bar plots (bottom row) show the frequency of binary variables: diabetes mellitus, history of stroke or transient ischemic attack (TIA), and anticoagulant use for atrial fibrillation. These categorical predictors demonstrate distinct group distributions, supporting their relevance in the assessment of cognitive risk in HFpEF.

Figure 3 illustrates the distribution of key predictors of cognitive impairment among HFpEF patients. Notably, KCCQ scores are generally high, with a substantial proportion of patients scoring above 60, indicating preserved self-reported quality of life. The eGFR values exhibit a moderate spread, reflecting variability in renal function within the cohort. NT-proBNP levels display a pronounced right skew, as expected given their known clinical variability and wide range in heart failure populations. The binary variables—diabetes mellitus, history of stroke/TIA, and anticoagulant use for atrial fibrillation—demonstrate clearly defined group distributions.

**Figure 4:**
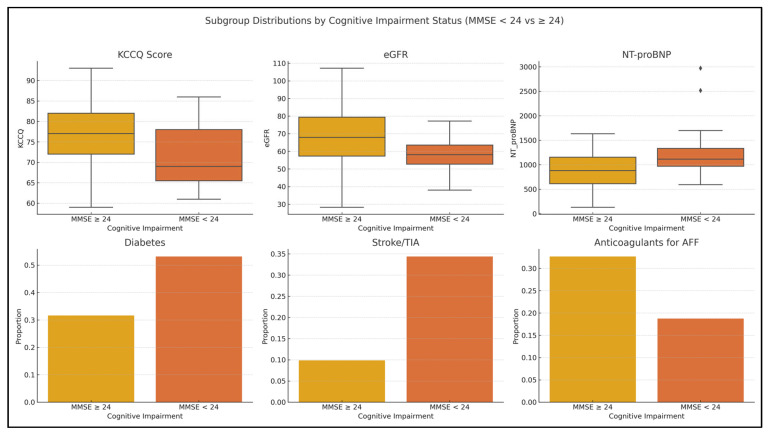
The distribution of key clinical predictors stratified by cognitive status (MMSE < 24 vs. ≥24) in HFpEF patients. Top row: Boxplots show continuous variables—KCCQ score, eGFR, and NT-proBNP. Patients with preserved cognition (MMSE ≥ 24) exhibit higher KCCQ scores and eGFR, while NT-proBNP levels are elevated and right-skewed in the cognitively impaired group (MMSE < 24). Bottom row: Bar plots represent the proportions of patients with diabetes mellitus, history of stroke/TIA, and use of anticoagulants for atrial fibrillation. Comorbidities are more frequent in the MMSE < 24 group, while anticoagulant use is higher among cognitively preserved patients, suggesting a potential protective effect.

The distribution of key clinical predictors stratified by cognitive status (MMSE < 24 vs. ≥24) is presented in Figure 4. Boxplots and bar charts illustrate how variables like KCCQ, eGFR, NT-proBNP, and key comorbidities differ by cognitive function. Patients with an MMSE ≥ 24 demonstrated higher Kansas City Cardiomyopathy Questionnaire (KCCQ) scores, indicating better perceived quality of life, and higher estimated glomerular filtration rate (eGFR) values, reflecting more preserved renal function. In contrast, NT-proBNP levels were elevated and right-skewed among cognitively impaired individuals, consistent with greater cardiac stress in this subgroup. The prevalence of diabetes mellitus and history of stroke or transient ischemic attack (TIA) was substantially higher in the MMSE < 24 group, highlighting the role of metabolic and cerebrovascular comorbidities in cognitive decline. Notably, the use of anticoagulants for atrial fibrillation was more common in cognitively preserved patients (MMSE ≥ 24), suggesting a potential protective association, possibly related to stroke prevention. These distributions align with multivariable regression findings and reinforce the clinical relevance of these predictors in cognitive impairment among HFpEF patients.

### 3.3. Cognitive Risk Score Development

To support the early identification of cognitive impairment (defined as MMSE < 24) among patients with heart failure with preserved ejection fraction (HFpEF), we developed a predictive scorecard based on multivariable logistic regression modeling. This scorecard, presented in Table 4, is intended for use as a screening tool and is not designed to replace comprehensive neurocognitive evaluation. Instead, it aims to guide clinicians in identifying patients who may benefit from further cognitive assessment or specialist referral.

**Figure 5:**
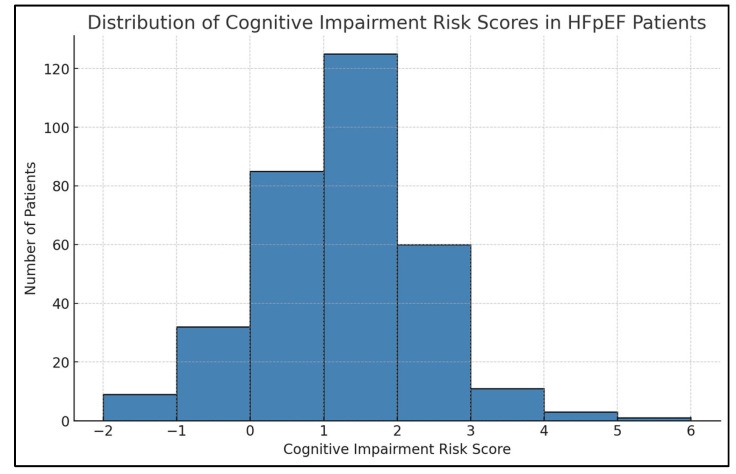
Distribution of cognitive impairment risk scores in HFpEF patients.

Figure 5 illustrates the distribution of cognitive impairment risk scores among HFpEF patients. Application of the scorecard to our cohort revealed that most patients scored between 0 and 2, indicating a moderate overall risk burden for cognitive impairment within this population. Fewer patients exhibited scores at the extreme low (≤−1) or high (≥4) ends, indicating less common profiles of either minimal or high cognitive risk. This distribution supports the clinical applicability of the scorecard as a screening tool across a broad HFpEF population.

**Figure 6:**
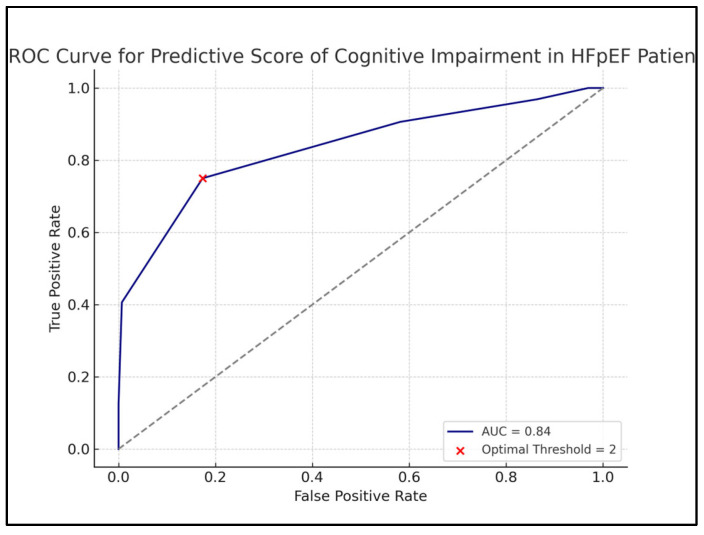
Receiver operating characteristic (ROC) curve evaluating the performance of the cognitive impairment risk score in predicting MMSE-2 scores < 24 among HFpEF patients.

The ROC curve demonstrates the performance of the cognitive impairment risk score, developed using multivariable logistic regression and validated against MMSE-2 scores < 24. The area under the curve (AUC) is 0.84, indicating the strong discriminative ability of the score to differentiate between patients with and without cognitive impairment (Figure 6).

Using a threshold score of ≥2, the model achieved an optimal balance between sensitivity (75%) and specificity (83%), identifying patients at higher risk of cognitive impairment. This threshold can aid clinicians in screening and determining which patients may benefit from further cognitive assessment or specialist referral.

## 4. Discussion

While several studies have previously examined cognitive impairment in patients with heart failure, most have focused on HFrEF populations or used limited cognitive screening tools. Our study differs in that it specifically targets elderly patients with HFpEF, incorporates a broader set of clinical, metabolic, and vascular variables, and uniquely includes patient-reported health status (12-KCCQ) as a protective factor. Com-pared to prior work such as the ESC Heart Failure Long-Term Registry and the PARA-GON-HF trial, which emphasized cardiac and vascular comorbidities, our findings ex-tend the understanding of cognitive risk by integrating quality of life and kidney function into the predictive model. Additionally, while earlier studies identified associations, few proposed practical risk stratification tools. Our study addresses this gap by developing and validating a simple point-based scorecard tailored for routine clinical use, particularly in underrepresented Eastern European populations, thus enhancing its practical relevance and originality.

This study highlights the intricate relationship between metabolic, vascular, and cardiac dysfunction and cognitive decline in elderly patients with heart failure with preserved ejection fraction (HFpEF). Cognitive impairment was highly prevalent in our cohort, and several independent clinical predictors were identified, including diabetes mellitus, history of stroke or transient ischemic attack (TIA), carotid artery disease, elevated NT-proBNP levels, lower estimated glomerular filtration rate (eGFR), and reduced health-related quality of life assessed by the Kansas City Cardiomyopathy Questionnaire (KCCQ). Importantly, anticoagulant therapy for atrial fibrillation was associated with a protective effect against cognitive decline.

Our findings align with previous research indicating that HFpEF patients are at heightened risk for cognitive impairment due to a combination of systemic vascular disease, metabolic abnormalities, and cardiac dysfunction [38,39,40,41,42]. Diabetes mellitus, a major metabolic risk factor, is known to accelerate cognitive decline through mechanisms such as microvascular injury, chronic inflammation, oxidative stress, and direct neuronal damage [20,21]. Similarly, the significant association of prior stroke or TIA and carotid artery disease with lower MMSE-2 scores emphasizes the contribution of cerebrovascular pathology to cognitive dysfunction [43,44].

Cardiac biomarkers such as NT-proBNP, traditionally used to assess heart failure severity, have been increasingly recognized as indicators of cerebral hypoperfusion and cognitive dysfunction [11,39]. In our study, elevated NT-proBNP levels were modestly but significantly associated with increased cognitive risk, supporting the hypothesis that chronic hemodynamic stress and neurohormonal activation adversely affect brain function.

Interestingly, a better quality of life as reflected by higher KCCQ scores was independently associated with lower cognitive risk. This observation underlines the important link between physical, functional, and cognitive health in HFpEF. Previous studies have shown that functional decline often parallels cognitive deterioration, suggesting that interventions aimed at improving physical status could potentially confer cognitive benefits [7,13].

The observed protective effect of anticoagulant use for atrial fibrillation is particularly notable. Atrial fibrillation is a known risk factor for embolic strokes and silent cerebral infarctions, both of which contribute to cognitive decline [45,46,47]. Anticoagulation likely mitigates this risk by preventing thromboembolic events, thus preserving cognitive function. This finding emphasizes the importance of rigorous rhythm management and anticoagulation strategies in HFpEF patients, not only to prevent stroke, but also to potentially protect cognitive health.

When comparing our results with those of previous studies, several important observations emerge. Similar to the findings by Faulkner et al. [22], who reported that diabetes and vascular disease are strong predictors of cognitive decline in HFpEF, our study confirms the prominent role of metabolic and vascular factors. Additionally, our results are consistent with the ESC Heart Failure Long-Term Registry [3], which emphasized the association between cognitive dysfunction and clinical comorbidities such as atrial fibrillation, diabetes, and chronic kidney disease in HF patients. However, our inclusion of the 12-KCCQ score as a protective factor provides new insight, reinforcing the suggestion from Goh et al. that functional status is an important but underrecognized determinant of cognitive health in HF [7]. Unlike some prior studies, which identified depression as an independent predictor of cognitive impairment, in our multivariable analysis, depression did not retain independent predictive value after adjusting for other vascular and metabolic factors. This discrepancy may reflect differences in study populations, assessment tools, or sample sizes. Our findings also complement the European Heart Failure Association position paper, which highlighted the role of brain–heart interactions mediated through vascular, inflammatory, and hemodynamic pathways [23].

When comparing our findings to prior large-scale studies such as PARAGON-HF, important similarities and differences emerge. In the PARAGON-HF trial, Shen et al. conducted a prespecified analysis examining cognitive function in HFpEF patients and identified that higher NT-proBNP levels, older age, and vascular comorbidities (including hypertension, atrial fibrillation, and prior stroke) were independently associated with worse cognitive performance [24]. Similarly, in our cohort, elevated NT-proBNP, history of stroke/TIA, and vascular disease (notably carotid artery disease) were strong predictors of cognitive impairment. This consistency underscores the importance of hemodynamic and vascular factors across different HFpEF populations. However, unlike PARAGON-HF, where global cognitive screening was limited and did not include quality of life or metabolic markers in the cognitive models, our study additionally identified diabetes mellitus, eGFR decline, and lower health-related quality of life (KCCQ) as independent risk or protective factors. These differences may reflect variations in study design, population characteristics, or the broader set of variables assessed. Our findings complement those of PARAGON-HF by emphasizing not only cardiac biomarkers, but also functional and metabolic health as critical components influencing cognitive status in HFpEF.

The development of a practical, point-based predictive scorecard integrating these variables offers a useful tool for clinical practice. By applying easily obtainable clinical and laboratory measures, clinicians can stratify HFpEF patients by their risk of cognitive impairment and prioritize cognitive screening or early interventions. Our model demonstrated strong performance, with an area under the ROC curve (AUC) of 0.84 and good sensitivity and specificity at a score threshold of 2. Such early identification is crucial, as cognitive impairment in HFpEF patients has been associated with impaired self-care, reduced medication adherence, more frequent hospitalizations, and higher mortality.

These findings are consistent with broader epidemiologic data showing that cognitive impairment is highly prevalent in HFpEF populations and may worsen outcomes [6,41]. However, few studies have proposed practical, validated tools for routine clinical use, especially in Eastern European populations where data are sparse. Our study adds important regional data and suggests that incorporating cognitive risk assessment into routine HFpEF care may be both feasible and impactful.

Nonetheless, this study has limitations. First, the cross-sectional design precludes any inference of causality between the identified predictors and cognitive impairment. Longitudinal studies are necessary to establish temporal relationships. Second, while the MMSE-2 is a widely used cognitive screening tool, it may lack sensitivity for detecting mild cognitive impairment or specific cognitive domains (e.g., executive function) particularly affected in HF. Comprehensive neuropsychological testing would provide a more detailed cognitive profile. Third, our cohort was drawn from a single center in Western Romania, and the generalizability to other populations with different demographic and clinical characteristics may be limited. Another limitation is that this study defined cognitive impairment as MMSE ≤ 23 (suspected dementia), but only 32 participants met this criterion, representing less than 10% of the sample. This class imbalance may have limited the statistical power and stability of the regression analysis. Given that 74 additional participants scored between 24 and 27 (suggestive of mild cognitive impairment), future analyses could consider a broader cutoff (MMSE ≤ 27) to more accurately capture cognitive decline and improve model robustness. Finally, although we internally validated our scorecard, external validation in independent cohorts is required before broader clinical implementation.

Future research should focus on validating this risk score externally, exploring whether early intervention based on risk stratification can prevent or slow cognitive decline, and investigating the biological mechanisms linking HFpEF and cognitive dysfunction. Additionally, it would be valuable to assess the impact of multidisciplinary interventions—targeting metabolic, vascular, and cardiac risk factors—on cognitive trajectories in this vulnerable patient population.

## 5. Conclusions

Cognitive impairment is common in elderly patients with heart failure with preserved ejection fraction (HFpEF) and is closely associated with modifiable vascular, metabolic, and cardiac factors. This study identified several independent predictors of cognitive decline, including diabetes, stroke or TIA history, carotid artery disease, reduced eGFR, elevated NT-proBNP levels, and lower quality of life as assessed by the 12-KCCQ. Notably, anticoagulant use for atrial fibrillation was associated with a protective effect. Based on these findings, we developed a novel, practical, point-based risk score that may support the early recognition of cognitive impairment in clinical settings. This tool has the potential to enhance cognitive screening, guide timely interventions, and improve overall care in HFpEF populations. Future studies should focus on external validation and integration into multidisciplinary heart failure management strategies.

## Figures and Tables

**Table 1:** Patient characteristics according to baseline MMSE score category.

Variable	MMSE Score Category			*p* for Trend
	I (28–30) n = 220	II (24–27) n = 74	III (<24) n = 32	
MMSE score	29.3 ± 0.7	25.1 ± 1.1	20.5 ± 2.9	<0.0001
Age, y	72.2 ± 5.4	73.4 ± 5.2	74.6 ± 6.3	<0.0001
Women	108 (49)	39 (52)	19 (59)	0.31
Systolic BP, mm Hg	128.4 ± 14.5	129.5 ± 15.7	130.2 ± 15.9	0.10
Diastolic BP, mm Hg	75.4 ± 5.9	72.8 ± 6.3	71.5 ± 5.9	<0.001
Pulse pressure, mm Hg	70.3 ± 6.3	70.2 ± 6.1	70.1 ± 4.9	0.44
Pulse rate, beats/min	80.5 ± 10.5	80.3 ± 10.7	80.8 ± 12.5	0.38
Body mass index, kg/m^2^	29.8 ± 3.8	29.9 ± 5.4	29.8 ± 4.9	0.40
NYHA class III/IV	33 (15)	10 (14)	5 (16)	0.84
12-KCCQ	77.3 (67.8–87.5)	75.7 (58.5–93.4)	74.8 (60.4–88.6)	0.043
LVEF, %	57.4 ± 7.3	56.9 ± 6.8	55.6 ± 4.9	0.37
Alcohol use	18 (8)	7 (9)	4 (12)	0.32
Current smoker	11 (5.0)	5 (6.7)	3 (9.3)	0.27
Previous HF hospitalization	90 (41)	33 (45)	15 (48)	0.44
Old MI	42 (19)	16 (22)	7 (24)	0.4
Angina pectoris	68 (31)	22 (30)	10 (32)	0.95
CABG or PCI	53 (24)	19 (26)	9 (27)	0.6
Hypertension	196 (89)	65 (88)	29 (90)	0.95
Diabetes	68 (31)	33 (44)	15 (48)	0.014
AFF history	119 (54)	41 (55)	14 (45)	0.2
AFF on ECG	64 (29)	23 (31)	10 (32)	0.72
Stroke or TIA	22 (10)	11 (15)	7 (21)	0.031
Carotid AD	11 (5)	6 (8)	5 (15)	0.015
PAD	13 (6)	6 (8)	3 (9)	0.37
Depression	13 (6)	10 (13)	5 (17)	0.015
COPD	31 (14)	9 (12)	4 (13)	0.69
eGFR, mL/min/1.73 m^2^	68.5 ± 14.5	65.3 ± 18.4	60,1 ± 9.2	<0.01
Hematocrit, %	42.7 ± 4.6	42.5 ± 4.8	41.8 ± 4.3	0.35
Hemoglobin, g/dL	13.6 ± 4.2	13.4 ± 4.5	13.1 ± 4.1	0.373
Hemoglobin A1c, %	5.6 ± 1.4	6.2 ± 1.5	6.9 ± 2,2	<0.001
Total cholesterol, mg/dL	210 ± 34	227 ± 36	224 ± 32	<0.001
Triglycerides, mgl/dL	178 ± 26	213 ± 25	244 ± 16	<0.001
Uric acid, mgl/dL	6.4 ± 0.3	6.5 ± 0.3	6.6 ± 0.2	<0.001
NT-proBNP, pg/mL	823 (250–1420)	1054 (481–1635)	1114 (593–2516)	<0.0001
Diuretics	204 (93)	70 (94)	29 (92)	0.82
Digoxin	18 (8.4)	7 (8.9)	3 (9.3)	0.73
ACEI or ARB	183 (83)	63 (85)	27 (84)	0.75
Beta-blockers	167 (76)	55 (74)	23 (73)	0.6
MRAs	123 (56)	40 (54)	15 (48)	0.37
CCB	103 (47)	38 (52)	16 (49)	0.55
Antiplatelets	163 (74)	56 (76)	23 (72)	0.93
Anticoagulants	75 (34)	21 (29)	6 (18)	0.036
Statins	185 (84)	58 (79)	26 (82)	0.4
Pacemaker	13 (6)	4 (5)	1 (4)	0.56

Data are presented as mean±SD, number (%), or median (interquartile range). KCCQ ranges from 0 to 100. Abbreviations: ACEI, angiotensin-converting enzyme inhibitor; AD, artery disease; AFF, atrial fibrillation or flutter; ARB, angiotensin receptor blocker; BP, blood pressure; CABG, coronary artery bypass grafting; CCB, calcium channel blockers; COPD, chronic obstructive pulmonary disease; eGFR, estimated glomerular filtration rate; HDL, high-density lipoprotein; HF, heart failure; KCCQ, Kansas City Cardiomyopathy Questionnaire; LVEF, left ventricular ejection fraction; MI, myocardial infarction; MMSE, Mini-Mental State Examination; MRA, mineralocorticoid receptor antagonist; NT-proBNP, N-terminal pro-B-type natriuretic peptide; NYHA, New York Heart Association; PAD, peripheral artery disease, TIA, transient ischemic attack.

**Table 2:** Univariate logistic regression results predicting cognitive impairment (MMSE < 24).

	Univariate Regression Analysis		
Variable	OR	95% CI	*p*-Value
Age	1.07	1.02–1.12	0.005
KCCQ	0.18	0.08–0.39	<0.001
Diabetes	2.44	1.17–5.11	0.01
Stroke/TIA	4.78	2.09–10.91	<0.001
Carotid artery disease	5.43	2.12–13.87	0.001
Depression	3.16	1.81–11.45	<0.01
eGFR mL/min/1.73 m^2^	0.95	0.93–0.98	<0.01
Total Cholesterol	1.01	0.65–1.59	<0.01
Triglycerides	1.02	1.01–1.09	0.03
Uric acid	2.44	2.13–8.90	0.04
NT-proBNP	1.02	1.0–1.03	<0.0001
Anticoagulants for AFF	0.47	0.18–1.19	<0.04

Footnote: Dependent variable: MMSE group; 0 = normal, 1 = mild cognitive impairment, 2 = suspected dementia). Abbreviations: KCCQ, Kansas City Cardiomyopathy Questionnaire; LVEF, left ventricular ejection fraction; MI, myocardial infarction; MMSE, Mini-Mental State Examination; NT-proBNP, N-terminal pro–B-type natriuretic peptide; AFF, atrial fibrillation or flutter; TIA, transient ischemic attack; eGFR, estimated glomerular filtration rate.

**Table 3:** Multivariable logistic regression results predicting cognitive impairment (MMSE-2 < 24).

Predictor	β Coefficient	Odds Ratio	*p*-Value
Anticoagulants for AFF	−3.487	0.0306	0.0017
Carotid artery disease	+2.255	9.5349	0.0106
12-KCCQ	−0.063	0.9392	0.0470
Diabetes	+1.698	5.4651	0.0095
Stroke/TIA	+1.790	5.9866	0.0349
eGFR	−0.050	0.9513	0.0044
NT-proBNP	+0.0023	1.0023	0.0028

Footnote: Logistic regression was performed to identify independent predictors of cognitive impairment, defined as MMSE-2 score < 24 (binary outcome: 1 = impaired, 0 = not impaired). Odds ratios (ORs) represent the likelihood of cognitive impairment per unit change in each predictor, holding other variables constant. Abbreviations: KCCQ, Kansas City Cardiomyopathy Questionnaire; LVEF, left ventricular ejection fraction; MI, myocardial infarction; MMSE, Mini-Mental State Examination; NT-proBNP, N-terminal pro-B-type natriuretic peptide; AFF, atrial fibrillation or flutter; TIA, transient ischemic attack; eGFR, estimated glomerular filtration rate.

**Table 4:** Predictive scorecard for cognitive impairment in elderly HFpEF patients.

Risk Factors and Assigned Point Values		
Predictor	Criteria	Points
12-KCCQ Score	≤70	+1
eGFR	<60 mL/min/1.73 m^2^	+1
NT-proBNP	≥900 pg/mL	+1
Diabetes	Present	+1
Stroke/TIA History	Present	+1
Carotid Artery Disease	Present	+1
Anticoagulants for AFF	Present	−2
Max Score = 6, Min Score = −2		
**Score Interpretation**		
**Total Score**	**Risk Interpretation**	
≤0	Low risk of cognitive impairment	
1–2	Moderate risk	
≥3	High risk of cognitive impairment (MMSE < 24)	
**Recommended Cutoff and Model Performance**		
Optimal cutoff: Score ≥ 2 At this cutoff: ○ **Sensitivity:** 75% ○ **Specificity:** 83% ○ **AUC:** 0.90 (ROC analysis)		

Footnote: Point values were assigned based on the β coefficients from the multivariable logistic regression model. Each variable included in the final model was selected based on clinical relevance and statistical significance (*p* < 0.05). Continuous variables were categorized using clinically meaningful cutoffs. The β coefficients were then scaled and rounded to the nearest whole number to facilitate practical application. Variables associated with increased cognitive risk were assigned positive values, while protective variables were given negative values. The total score reflects the cumulative cognitive risk in elderly HFpEF patients, with higher scores indicating greater likelihood of MMSE-2-defined cognitive impairment. Abbreviations: HFpEF, heart failure with preserved ejection fraction; AFF, atrial flutter/fibrillation; TIA, transient ischemic attack; eGFR, estimated glomerular filtration rate; KCCQ, Kansas City Cardiomyopathy Questionnaire; MMSE, Mini-Mental State Examination; NT-proBNP, N-terminal pro-B-type natriuretic peptide; AUC, area under the curve; ROC, receiver operating characteristic.

## Data Availability

The data are available upon request to the first author, S.-F.A.
